# New Insights into the Role of the Immune Microenvironment in Breast Carcinoma

**DOI:** 10.1155/2013/785317

**Published:** 2013-06-03

**Authors:** Luis de la Cruz-Merino, Antonio Barco-Sánchez, Fernando Henao Carrasco, Esteban Nogales Fernández, Ana Vallejo Benítez, Javier Brugal Molina, Antonio Martínez Peinado, Ana Grueso López, Manuel Ruiz Borrego, Manuel Codes Manuel de Villena, Víctor Sánchez-Margalet, Adoración Nieto-García, Emilio Alba Conejo, Noelia Casares Lagar, José Ibáñez Martínez

**Affiliations:** ^1^Clinical Oncology Department, Hospital Universitario Virgen Macarena, Avenida Dr. Fedriani s/n, 41009 Sevilla, Spain; ^2^Biochemistry Department, Hospital Universitario Virgen Macarena, Avenida Dr. Fedriani s/n, 41009 Sevilla, Spain; ^3^Pathology Department, Hospital Universitario Virgen Macarena, Avenida Dr. Fedriani s/n, 41009 Sevilla, Spain; ^4^Clinical Oncology Department, Hospital Universitario Virgen del Rocío, Avenida Manuel Siurot s/n, 41013 Sevilla, Spain; ^5^Statistics Department, Universidad de Sevilla, Avenida Dr. Fedriani s/n, 41009 Sevilla, Spain; ^6^Clinical Oncology Department, Hospital Universitario Virgen de la Victoria, Campus de Teatinos s/n, 29010 Málaga, Spain; ^7^Centro de Investigación Médica Aplicada (CIMA), Universidad de Navarra, Pío XII 55, 31008 Pamplona, Spain

## Abstract

Recently, immune edition has been recognized as a new hallmark of cancer. In this respect, some clinical trials in breast cancer have reported imppressive outcomes related to laboratory immune findings, especially in the neoadjuvant and metastatic setting. Infiltration by tumor infiltrating lymphocytes (TIL) and their subtypes, tumor-associated macrophages (TAM) and myeloid-derived suppressive cells (MDSC) seem bona fide prognostic and even predictive biomarkers, that will eventually be incorporated into diagnostic and therapeutic algorithms of breast cancer. In addition, the complex interaction of costimulatory and coinhibitory molecules on the immune synapse and the different signals that they may exert represent another exciting field to explore. In this review we try to summarize and elucidate these new concepts and knowledge from a translational perspective focusing on breast cancer, paying special attention to those aspects that might have more significance in clinical practice and could be useful to design successful therapeutic strategies in the future.

## 1. Introduction

Neoplasms represent a wide group of heterogeneous diseases with several different alterations at genomic and proteomic levels, which finally confer them the acquisition of the neoplastic phenotype. Every human carcinoma induces an immune response in its microenvironment. Generally, this immune reaction is considered ineffective to destroy cancer cells; however, in the last years evidence has emerged demonstrating the importance of tumor lymphocyte infiltration in the clinical evolution of many cancer types. Importantly, this immune “awakening” against tumors may be induced by some new and classical antineoplastic strategies. Hence, we will analyze this new knowledge from a clinical point of view focusing on breast cancer, giving eventual clues to overcome and break immune tolerance in this disease.

## 2. Clinical Consequences of Immune-Related Events in Breast Carcinoma

In the last few years, some translational studies in patients with breast carcinoma have suggested that infiltration by tumor infiltrating lymphocytes (TIL) and regulatory T (Treg) cells might have a great significance in the final clinical outcomes. 

In the neoadjuvant setting Demaria et al. found a change in the frequency of TIL after treatment with paclitaxel. Importantly, response was correlated with TIL density suggesting that apoptosis induced by taxanes is a powerful immunogenic stimulus [1].

Recently, Denkert et al. investigated the hypothesis that the presence of an intense lymphocytic infiltrate might predict the response to neoadjuvant chemotherapy in breast cancer [2]. They examined pretherapeutic core biopsies of 1058 patients enrolled in the GeparDuo and GeparTrio studies. Results of these analyses showed that the presence of intratumoral lymphocytes and lymphocyte-predominant breast cancers were associated with a 31 and 41% pathological complete response (pCR) rates, respectively [2]. On the opposite, pCR rates were only 2% in patients without any lymphocytic infiltration. In a multivariate analysis, intratumoral lymphocytes, age, and estrogen receptor status were the only independent predictive parameters for pCR. Several other studies have reported consistent data in the same direction [3]. These results should not be overlooked as they confirm a strong association between lymphocytic infiltrate and chemotherapy response in a large set of more than 1000 samples of breast cancer.

Despite the impressive results achieved in the study conducted by Denkert, TIL subtypes were not specifically analyzed. On the contrary, Ladoire et al. reported another interesting study conducted on 56 patients with operable breast carcinoma and treated with preoperative chemotherapy [4]. Overall, the histological analyses of surgical specimens revealed a pathologic complete response in 21.4% of the cases. pCR was achieved in 40% of the tumors overexpressing HER2 and only in 11% of the patients with HER2-negative tumors. Results of T-cell infiltrates analyses were of great interest at this point. After neoadjuvant chemotherapy CD3 and CD8 infiltrates remain stable, whereas FOXP3+ Treg cell numbers significantly decreased in surgical specimens. Importantly, pCR patients had a significantly lower number of FOXP3 cells than nonresponders [4]. Accordingly with this study, Perez et al. found that advanced breast cancer patients with HER2+ tumors exhibited an overall significantly increased frequency of circulating Treg, and, among them, therapeutic intervention with trastuzumab led to an overall reduction to normal levels in the frequency of Treg [5]. Remarkably, a good clinical response to trastuzumab therapy was associated with a significant reduction in Treg frequency, whereas disease recurrence correlated with a significant increase in the percentage of circulating Treg [5]. 

Recently Mahmoud et al. analyzed the influence of density of CD8+ cytotoxic lymphocytes on prognosis in a large series of 1334 patients with primary invasive breast carcinomas. CD8+ T cells were counted in three locations per tumor (intratumoral compartment, adjacent, and distant stroma), and the total number was determined by the sum of the counts of these three compartments. Higher total CD8+ lymphocyte counts were independently associated with longer breast cancer specific survival after multivariate analysis (Hazard Ratio, 0.55; 95% CI, 0.39-0.78; *P* = 0.001) in a model that included the standard prognostic and predictive factors [6].

Taking into account the above mentioned studies (Table 1), it is tempting to speculate that TIL infiltration may represent not only a prognostic but rather a predictive factor of clinical response in breast cancer, although prospective validation studies are needed to confirm this hypothesis.

## 3. Regulatory T Lymphocytes and Host Antitumor Response

Tumor infiltration by TIL is well recognized as a good prognosis factor in multiple solid human neoplasms [7–10]. TIL are considered to be a manifestation of the host antitumor reaction. However, there is growing evidence that the specific type of immune cells rather than their quantity governs the host-versus-tumor immune response. 

The majority of TIL in solid tumors is CD3+ T-cell phenotype. CD3 can be divided into CD4+ helper cells, including Th1 and Th2 subtypes, based on their cytokine profile, CD4+ regulatory T cells (Treg), and CD8+ cytotoxic effector cells. Treg are thymus-derived CD4+CD25+ T lymphocytes that constitutively express cell surface cytotoxic T lymphocyte antigen-4 (CTLA-4) and secrete immunosuppressive cytokines such as TGF-beta and IL-10 [11]. Treg may also be induced in the periphery from naïve T cells under certain conditions, like stimulation with TGF-*β* [12]. 

Treg represent roughly 10% of CD4 T cells and specifically express the forkhead box P3 transcription factor (FOXP3) [13, 14]. Recent studies have shown that Treg play an essential role in sustaining self-tolerance by expressing a wide variety of pathological immune responses against self, nonself, and tumor antigens [11]. Although the exact mechanisms of Treg suppression remain unknown, this effect seems to be largely dependent on the expression of the transcription factor FOXP3 which controls some genes encoding proteins like CD25, GITR, CTLA-4, and others, capable of mediating Treg suppressive functions [15, 16]. In addition, FOXP3 inhibits production of effector cytokines like interleukin-2 (IL-2) after T-cell receptor (TCR) stimulation of T cells [17]. Other mechanisms of immunosuppression are direct cell-to-cell contact with antigen presenting cells (APC) via transforming growth factor *β* (TGF-*β*) or CTLA-4 and secretion of immunosuppressive cytokines such as interleukin-10 (IL-10), TGF-*β*, and others [18, 19]. 

Specifically in breast carcinoma, the number of FOXP3 CD4+CD25+ Treg and decreased ratios of CD8 T cells/FOXP3 Treg are correlated with a poor prognosis [20]. Bates et al., after analyzing 222 breast carcinoma specimens, observed that elevated numbers of Treg confer a significantly shorter overall and recurrence-free survival and that Treg quantity correlates significantly with more aggressive breast cancer features (high tumor grade, node positive disease) [20]. Although FOXP3 expression was thought to be restricted to Treg, recently it has been elucidated that this transcription factor is also present in various types of tumor cells, including breast cancer [21–23]. However, clinical implications of FOXP3 expression in breast cancer cells are contradictory. Balsari et al. reported that accumulation of FOXP3 Treg predicts a striking reduction of patient survival [21]. On the contrary, Ladoire et al., in a large prospective cohort of node positive breast cancer included in the PACS01 trial, have recently reported better outcomes for those patients that express FOXP3 in breast cancer cells (37% of the entire population) but only in the group treated without taxanes [22]. The same group has published data of a retrospective study restricted to a HER2-positive population treated with neoadjuvant chemotherapy [23]. Again in this case FOXP3 expression in tumor cells correlates with better relapse free and overall survival. In line with these results, FOXP3 has been recently demonstrated to be a tumor suppressor gene that acts as a transcriptional repressor of some breast cancer oncogenes [23].

 As mentioned before, immune function is generally compromised in cancer patients, which have lower absolute numbers of peripheral blood lymphocytes but increased numbers of functionally suppressive CD4+CD25+ Treg and dysfunctional dendritic cells (DC) in peripheral blood and tumor microenvironment [24]. In addition, higher numbers of Treg in blood from patients with breast cancer have been reported in relation to normal donors [25]. 

## 4. Mechanisms of Immune Tolerance: Effects of Costimulatory and Coinhibitory Molecules on the Immune Synapse

The immune synapse is a region of physical contact between the T cell and the antigen presenting cell (APC), and it represents one of the major determinants of the immune response against tumoral antigens [26]. Two main signals are required for an effective T-cell activation. The first signal is provided by the recognition of cognate antigen bound major histocompatibility complex (MHC) by the T-cell receptor (TCR) [27]. Additional costimulatory signals are provided by engagement of coreceptors. The canonical coreceptor CD28 binds to members of the B7 family present on APC. However, soon after T-cell priming, other negative regulatory molecules are induced on T-cells leading to downregulation of the T cell response. Some of the main costimulatory and coinhibitory molecules that act as immune checkpoints on the immune synapse are resumed in the following lines.

**Figure 1:**
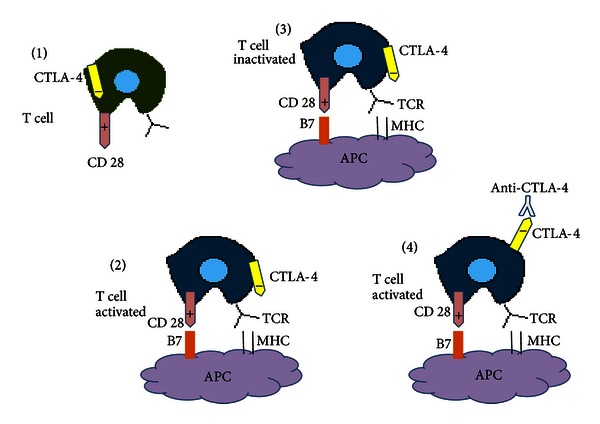
Role of CTLA-4 in T-cell activation. (1) CTLA-4 is a negative regulator of T-cell activation. (2) Conventional T cells are activated by engagement of MHC and B7. (3) Upon activation, T-cells express CTLA-4. Binding of CTLA-4 with B7 inhibit T cell activation. (4) Blockade of CTLA-4 produces the liberation of CD28 that engages with B7 activating T cells. APC: antigen presenting cell; MHC: major histocompatibility complex; and TCR: T-cell receptor.

- CD40: CD40 is a member of the tumor necrosis factor receptor family expressed on macrophages, dendritic cells, endothelial and B cells, and fibroblasts [28]. Binding of CD40 with its CD40 ligand (CD40L) or CD154 acts on APC and T cells mediating both cellular and humoral responses. Specifically on APC, CD40 plays a central role in priming and expansion of antigen-specific CD4 T cells by regulating the expression of costimulatory molecules on APC such as CD80 and CD86 (B7.1 and B7.2) and by production of cytokines such as IL-12, IL-8, or TNF-*α* [28, 29].
- Cytotoxic T lymphocyte antigen-4 (CTLA-4): CTLA-4 acts as a key negative regulator of CD28 dependent T-cell activation to limit self-damage [30]. CTLA-4 is produced and mobilized from the internal side of the cell membrane, to the immune synapses 2 to 3 days after T-cell activation has taken place. There, it is bound to either one of the costimulatory molecules, CD80 and CD86. CTLA-4 expression turns the activated T cell to an inhibitory T cell [31]. A delay in CTLA-4 expression favours T-cell activation and could be a pathway to improve or expand the immune response against tumors (Figure 1). Recently, an interesting study analyzed the effect of CTLA-4 in breast carcinoma [32]. CTLA-4 expression was detected in breast tissue and blood of breast cancer patients and normal donors. Patients showed strong expression of CTLA-4 in tumor cells of all specimens. By contrast, weakly positive or negative expression of CTLA-4 was found in normal breast tissue. In addition, patients with higher mRNA level of CTLA-4 had breast cancer with worse features, and spontaneous expression of CD3+CTLA-4+ on peripheral blood of patients with tumors was also significantly higher than that of the controls [32].
- Programmed death 1 (PD-1): PD-1 is expressed on activated T and B cells, natural killer, dendritic cells, and activated monocytes [33]. PD-1 plays a major role in maintenance of T-cell tolerance limiting effector T-cell responses. There are two ligands of PD-1, PD-L1 and PD-L2 (or B7-H1 and B7-H2), although PD-L1 is considered the most important one [34]. PD-L1 is aberrantly expressed in some tumors including breast cancer, and thus it can induce immune suppression through signaling PD-1 [35]. In breast cancer PD-L1 expression (in tumor tissue and TIL) has been shown to be correlated with worsen clinicopathological data like larger tumor size, histologic grade III tumors, or negative hormone receptors [36].
- OX-40: OX-40 is a member of the tumor necrosis factor (TNF) superfamily that needs T-cell activation to be expressed [37]. OX-40 is present in CD4+ and CD8+ T cells, whereas its ligand OX40L is expressed on activated APC, B cells, and macrophages [38]. Binding of OX40 to the OX40L enhances proliferation and survival of T cells leading to a larger expansion of effector T and a larger pool of memory T cells [37]. In addition, CD40 signaling increases cytokine secretion by CD4+ T cells and enhances the development of Th1 and Th2 cells [39].

The immune synapse is to be considered altogether as a complex battlefield where many different molecules and cells interact. It seems crucial to understand in depth the mechanisms that may trigger a successful immune response or on the contrary lead to immunotolerance at this level, in order to find out emerging therapeutic tools targeting the immune synapse.

## 5. Tumor-Associated Macrophages, Myeloid-Derived Suppressor Cells, and Related Cytokines

Chronic inflammation in some tissues correlates with higher risk of developing cancer [40]. Within the tumoral microenvironment, tumor-associated macrophages (TAM) and myeloid-derived suppressive cells (MDSC) seem to play a critical role in the progression of tumoral development through nonimmune (mostly proangiogenic) and immune mechanisms [41]. 

TAMs are a heterogeneous population of cells depending on oxygen availability and phases of tumor development [42]. In early stages, tumors are generally infiltrated by type 1 macrophages (M1) that release proinflammatory cytokines and chemokines promoting Th17 cell differentiation from naïve CD4+ T cells [43]. On the other hand, in advanced stages, TAMs polarize to a type 2 macrophage (M2) related cell that releases cytokines such as transforming growth factors *β*1 (TGF *β*1) and IL-10, which induce Th2 differentiation and recruitment, favouring Treg development and thus promoting tumor development through inhibition of anticancer immune responses [44]. 

In breast cancer, a sample of 128 matching invasive (88% stages I-II) and ductal carcinomas in situ specimens, along with normal breast tissues, was analyzed regarding macrophage phenotype [45]. Increased M2-TAM was significatively associated with more aggressive histopathological features (high tumor grade), increased microvessel density, and decreased overall survival, whereas M1-TAM phenotype was not associated with a worse overall survival. Furthermore, M1-TAM tumors were predominantly low grade [45].

MDSC represent a heterogeneous population of immature myeloid cells in different stages of myeloid cell differentiation [46]. MDSC within the tumor microenvironment exert a variety of immune suppressive functions by perturbing both innate and adaptive immune responses. These effects are largely dependent on cytokines (TGF-*β*, IL-10, and IL-6) and cellular factors observed in the surroundings of the tumors [47]. Recently, Cole et al. demonstrated that circulating MDSC in metastatic breast cancer significantly correlate with overall survival, observing worse outcomes in patients with high percentages of MDSC (OS, 6.9 versus 19.6 months; *P* = 0.05) [48]. 

These data suggest that MDSC might be a good biomarker and even a therapeutic target in breast cancer.

As previously cited, cytokines are molecules of critical importance in the tumor microenvironment that modulate the activity of immune cells and may induce different effects during tumor progression. Among immunosuppressive cytokines the role of TGF-*β* and IL-10 merits special consideration. Functions of both cytokines are intriguing and complex as long as they seem to play initially and antitumor effect preventing angiogenesis and inflammation by inhibiting macrophage activation [43]. Nevertheless, in established tumors, their effects are mostly protumorigenic, encouraging cell survival, and suppressing effector T cells [43]. Specifically, TGF-*β* plays a central role in the generation and function of CD4+CD25+ Treg and suppression of IFN-*γ* production by Th1 and CD8+ T cells, finally impeding a successful immune response and favouring tumor progression [19, 49, 50].

However, there are also cytokines in the microenvironment like GM-CSF or IL-2 that exert costimulatory effects [31]. GM-CSF has pleiotropic properties, including the mobilisation, differentiation, and function of dendritic cells [51], and it has also been studied in the clinical setting in many cancer types showing some promising results [52–54]. In this sense, Honkoop et al. reported an interesting study in locally advanced breast carcinoma treated with chemotherapy and GM-CSF [54]. The authors reported a positive correlation between the number of cycles received with GM-CSF and overall survival (OS) and disease-free survival (DFS) [54]. One of the hypothesis to explain these results relies in the large number of overexpressed tumoral antigens (Her-2/neu, CEA, MUC-1, etc.) in breast cancer released after CT, which represent an excellent target for an immune environment boosted by GM-CSF [55].

## 6. Strategies to Overcome Immunotolerance in Breast Cancer

### 6.1. Depletion of Treg

Depletion of FOXP3 Treg can enhance antitumor immunity, and thus different strategies are being pursued to attenuate the suppressive function of Treg [11]. 

Metronomic chemotherapy consists in administration of low doses of chemotherapeuticals with the aim of reducing tumor angiogenesis. Recently, it has been elucidated that low doses of oral metronomic cyclophosphamide in advanced cancer patients induce a profound and selective reduction of circulating regulatory T cells, associated with a suppression of their inhibitory functions on conventional T and NK cells, therefore, leading to reduction of tumor-induced immune tolerance and a better disease control [56, 57]. 

Treg are highly IL-2 dependent for their survival. IL-2 neutralization with specific antibodies may substantially reduce the number of Treg [58]. Denileukin diftitox (Ontak), a recombinant fusion protein consisting of IL-2 and diphtheria toxin, may deplete Treg and so reduce immune suppression boosting antitumor immunity [58, 59]. CD25 is the IL-2 receptor *α* chain, so denileukin diftitox binds to the IL-2 receptor and inhibits protein translation following internalization, leading to apoptosis [59]. Curiel demonstrated in a phase 0/1 trial that Ontak at 9 or 12 *μ*g/kg decreased the number of blood Treg and the suppression mediated by the CD4+CD25+ blood T-cell population in patients with advanced stage epithelial carcinomas, including cases of breast carcinoma [60].

### 6.2. Immune Synapses as a Therapeutic Target

As mentioned before, the immune synapses are virtual spaces where the complex controls and checkpoints that modulate the interaction of effector cells with their targets take place. Many of the molecules and cells that compose the immune synapses are attractive targets to be exploited clinically, and among them CTLA-4 is probably the most widely studied molecule. 

In preclinical studies with knockout mice it has been reported that CTLA-4 deficiency in CD4+CD25+ Treg impairs its suppressive function in tumor immunity [61, 62]. In addition, exclusive blockade of CTLA-4 signal in either CD4+CD25+ Treg or nonTreg T cell in mice leads not only to attenuation of Treg suppression but to augmentate effector T-cell activity [61]. 

A recent study demonstrated that antibodies against CTLA-4 (anti-CTLA-4) induce proliferation of TCR stimulated T effector cells and abrogate Treg suppressive activity by enhancing IL-2 and IFN*γ* release in response to polyclonal or tumor antigen stimulation [63]. Curiously, anti-CTLA-4 does not reduce the amount of Treg, suggesting that it mediates immune responses by direct activation of T effector cells and not by depleting Treg [63].

There are 2 CTLA-4 blocking antibodies for use in humans [64]. Recently, ipilimumab (Bristol-Myers Squibb, Princeton, JC, USA) has demonstrated significant benefits in overall survival in randomized phase III studies in the first or second line treatment of metastatic melanoma [65, 66], gaining FDA approval. 

Clinical research of anti-CTLA-4 in other solid neoplasms like breast carcinoma is scarce until now [67]. However a phase I study in advanced breast carcinoma with the combination of exemestane and tremelimumab has been recently reported, demonstrating that the combination is well tolerated and associated with an increased expression of inducible costimulator (ICOS+) in peripheral T CD4+ and CD8+ cells, which likely signals immune activation secondary to CTLA-4 blockade [68].

A better understanding of the mechanism of action of anti-CTLA-4, along with its use in the context of combinatorial strategies, may enable us to explore the eventual efficacy of these molecules in nonmelanoma populations [67, 69]. Combination of CTLA4 and PD-1 blockade with anti-PD-1:B7-H1 monoclonal antibodies increases effector T-cell infiltration into B16 melanoma in mice, resulting in an elevated effector to Treg cell ratio within the tumor [70]. Phase I studies in humans with single agent anti-PD-1 in refractory solid tumors have been performed with promising results [71].

As previously cited, CD40 is another molecule that plays an essential role in the immune synapses [35]. Several agonistic antibodies against CD40 are under clinical research, and preliminary data in murine models suggest strong immune effects resulting in CD4 T-cell priming and cytotoxic T-cell responses [72, 73]. Interestingly, a clinical study in 21 patients with advanced pancreatic carcinoma has been recently reported testing the combination of gemcitabine with CD40 agonist CP-870.893 [74]. Metabolic evaluation by PET assessment revealed an impressive response rate of 88% after two cycles, with a median progression free and overall survival of 5.6 and 7.4 months, respectively [74]. These interesting results deserve confirmation in phase II and III studies. Again, preclinical data have revealed that anti-CTLA-4 and CD40 are more effective when combined that either therapy alone [75].

Finally, another immunogenic molecule is OX40. It is elucidated that signaling through OX40 and OX40L enhances antitumor immunity [37]. In rodents, Murata et al. demonstrated that combination of a GM-CSF secreting tumor cell vaccine with anti-OX40 antibody induced a potent CD8+ T-cell response, leading to eradication of established breast carcinomas [76]. This effect seems related to the prolonged expansion and survival of tumor specific T cells. Another interesting strategy relies in the combination of OX40 therapy with radiotherapy (RT) and/or chemotherapy. CT and RT imply an enhanced expression of tumoral antigens with an increase in tumor antigen-specific cytotoxicity and OX40 expression. Clinical trials testing this hypothesis are ongoing [77, 78].

Although early in their clinical development, it is tempting to speculate that the universal mechanism of action of anti-CTLA-4, anti-PD-1, and CD40 or OX-40 agonists, among other molecules, may not only be restricted to melanoma patients but rather may be useful in a wide range of other oncologic diseases. Preclinical data support clinical research in this field especially in the context of combinatorial strategies. Likewise clinicians must be aware that conventional response criteria seem no longer valid in this context, and new guidelines for the evaluation of immune-related responses must be considered [79]. 

### 6.3. Impact of Chemotherapy (CT) in Breast Cancer Microenvironment

Chemotherapy remains the therapeutical modality of choice for the systemic treatment of many breast carcinomas, especially in the neoadjuvant and metastatic setting. Impact of conventional chemotherapy on the relationship between the tumor and the immune system is extremely important (Table 1). Some groups argue that cell death induced by chemotherapy implies a variety of immune reactions that mediate a sort of vaccination effect via release of an “antigenic milieu” that, in turn, may represent the major determinants of the therapeutical success of the chemotherapy in oncological diseases [80].

Cytotoxic drugs destroy tumor cells by apoptosis [57], and recent studies suggest that some chemotherapeutics may induce tumoral destruction improving cancer cell recognition by the immune system [81, 82]. Some preclinical studies support the idea that immune stimulation might be mediated by chemotherapy in murine cancer models treated with gemcitabine and doxorubicin [83, 84]. The explanation to this selective immune activation is an increased CD8 T-lymphocyte expansion and an increased density of TIL mediated by an effective MHC class I cross-presentation of tumor antigens released and phagocytosed [85]. 

**Figure 2:**
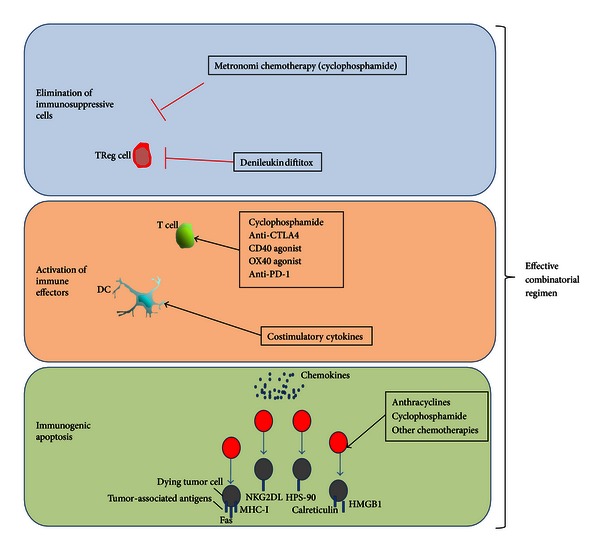
Mechanisms of action of conventional antineoplastic agents and new immunostimulatory drugs.

There is now clear evidence supporting the fact that drugs like anthracyclines, cyclophosphamide, or gemcitabine may promote apoptosis in cancer cells with immunogenic effects through several mechanisms [80, 85, 86] (Figure 2). This sort of immunogenic tumor cell death is characterized by a temporal sequence of events including early translocation of calreticulin (CRT) to the cell surface and thereafter interaction of CRT with multiple receptors on DC with apoptotic bodies phagocytosis, release and exposure of heat shock proteins, and late release of HMGB1 [85]. HMGB1 is able to bind to the TLR4 receptor on DC, which allows tumor-derived antigens to be processed and presented along with MHC and costimulatory molecules on the surface of DC [58, 62]. These mechanisms altogether serve to trigger DC-mediated specific antitumor response, which may be enhanced by the use of costimulatory molecules [31].

In addition, other more general effects of chemotherapy on the surrounding stroma are postulated like secondary necrosis or eradication of tumor cells [87]. Furthermore, gemcitabine has demonstrated the ability to restore immune surveillance by reducing MDSC levels in murine models, which represents another interesting field to explore among the immune effects of chemotherapy over microenvironment [88].

In conclusion, emerging evidence led Lake and Robinson to announce a paradigm shift in the way of understanding the effects of CT on the surrounding stroma [87, 89]. These new concepts may serve to consider chemotherapeutics as less empirical and more specific drugs, thus may help to customize treatments taking into account its potential effects on the microenvironment. 

## 7. Conclusions

Available data support the hypothesis of an immune-mediated antitumor activity in breast carcinoma, and several lines of research are ongoing [90]. It is critical to understand what happens in the tumoral microenvironment in order to design biological agents that may modulate the immune response towards cancer cell destruction (Figure 2). Combination strategies of chemoimmunotherapy will eventually synergize and obtain meaningful clinical results. 

Finally, the fact that probably the most successful strategy in oncology in the last decade has been the combination of chemotherapy and passive immunotherapy merits special consideration. Rituximab and trastuzumab monoclonal antibodies (MoAb) have obtained impressive results when administered to the right populations [91, 92]. Interestingly enough, the powerful effect of these MoAb is enhanced when combined with chemotherapy, as a new evidence of the priming of the APC tumor antigen presentation and T-cell activation. Moreover, preclinical data exploring the effects of a HER2/neu peptide vaccine combined with trastuzumab have demonstrated synergistic immune enhancement [93]. 

In our opinion there is large amount of available data which provides sufficient evidence to consider the host immune reaction as one of the main determinants of the clinical evolution in breast cancer. Importantly, this immune response is capable of being modulated in clinical practice, so new therapeutical strategies based on chemoimmunotherapeutic approaches might be worthy of consideration in the coming future to rise another step in the global battle against breast cancer.

## Figures and Tables

**Table 1:** Studies correlating immunobiomarkers with clinical results.

Study	*N* (patients)	Immune biomarker	Results
Balsari et al. [21]	DCIS: 62 Invasive: 257 Normal breast: 10	FOXP3	High FOXP3 in invasive and in situ breast carcinoma than in normal breast High FOXP3 shorter PFS and OS Negative correlation between FOXP3 and ER
Ladoire et al. [4]	56	CD3 CD8 FOXP3	Poor prognostic factors (RE−, high-tumor grade and nodal involvement) correlate with higher number of FOXP3 before chemotherapy >pCR to neoadjuvant chemotherapy correlates with absence of FOXP3 cells and presence of high number of CD8 T cell
Bates et al. [20]	183 + 214	FOXP3	FOXP3 expression in tumor associated with worse overall survival FOXP3 prognostic factor for distant metastases free survival
Demaria et al. [1]	25	TIL	Development of TIL after treatment correlates with clinical response to neoadjuvant chemotherapy
Denkert et al. [2]	1058 (2 cohort)	TIL	High TILs: pCR rates 42 and 40% versus 3 and 7%
Perez et al. [5]	24 normal breast 74 breast cancer (28 HER−; 46 HER+)	Tregs	Treg frecuency in HER2+ was significantly increased. Trastuzumab therapy: decreased Treg frecuency/objective clinical response
Mahmoud et al. [6]	1334	CD8+ T	TIL CD8+ density associated with improved clinical outcome

PFS: progression free survival; OS: overall survival; ER: estrogen receptor; and pCR: pathologic complete response.
